# Characterization and necrosis-inducing activity of necrosis- and ethylene-inducing peptide 1-like proteins from *Colletotrichum australisinense*, the causative agent of rubber tree anthracnose

**DOI:** 10.3389/fmicb.2022.969479

**Published:** 2022-08-30

**Authors:** Xianbao Liu, BoXun Li, Jimiao Cai, Yang Yang, Yanli Feng, Guixiu Huang

**Affiliations:** Chinese Academy of Tropical Agricultural Sciences, Environment and Plant Protection Institute, Key Laboratory of Integrated Pest Management on Tropical Crops, Key Laboratory for Monitoring and Control of Tropical Agricultural Pests, Ministry of Agriculture, Haikou, China

**Keywords:** *Colletotrichum acutatum* species complex, *Colletotrichum australisinense*, necrosis-and ethylene-inducing-like protein, rubber tree anthracnose, necrosis-inducing activity, pathogenesis, structural characterization

## Abstract

*Colletotrichum australisinense*, a member of the *Colletotrichum acutatum* species complex, is an important pathogen causing rubber tree anthracnose. Genome-wide comparative analysis showed this species complex contains more genes encoding necrosis- and ethylene-inducing peptide 1-like proteins (NLPs) than other *Colletotrichum* species complexes, but little is known about their necrosis-inducing roles in host. The aim of this study was to analyze NLPs number and type in *C. australisinense*, and characterize their necrosis-inducing activity in host or non-host. According to phylogenetic relationship, conserved the cysteine residues and the heptapeptide motif (GHRHDWE), 11 NLPs were identified and classified into three types. Five of the eleven NLPs were evaluated for necrosis-inducing activity. CaNLP4 (type 1) could not induce necrosis in host or non-host plants. By contrast, both CaNLP5 and CaNLP9 (type 1) induced necrosis in host and non-host plants, and necrosis-inducing activity was strongest for CaNLP9. CaNLP10 (type 2) and CaNLP11 (type 3) induced necrosis in host but not non-host plants. Substitution of key amino acid residues essential for necrosis induction activity led to loss of CaNLP4 activity. Structural characterization of CaNLP5 and CaNLP9 may explain differences in necrosis-inducing activity. We evaluated the expression of genes coding CaNLP by reverse transcription polymerase chain reaction (RT-PCR) and quantitative real-time PCR (qRT-PCR) at different time-points after pathogen infection. It was found that genes encoding CaNLPs with different activities exhibited significantly different expression patterns. The results demonstrate that CaNLPs are functionally and spatially distinct, and may play different but important roles in *C. australisinense* pathogenesis.

## Introduction

The genus *Colletotrichum* includes many plant pathogens that cause severe diseases affecting many economically important crops worldwide (Silva et al., 2017). *Colletotrichum* species exhibit different lifestyles and biotrophic, necrotrophic, and hemibiotrophic invasion strategies, of which hemibiotrophy is the most common. Pathogens utilize sequential biotrophic and necrotrophic infection strategies to invade and colonize host plants, and the switching process involves various virulence factors such as carbohydrate-active enzymes, secondary metabolites, secreted proteinases, and other types of effectors (O’Connell et al., 2012; Gan et al., 2013). The functions of effectors are closely related to the nutrition/infection strategies of pathogens (Villa-Rivera et al., 2017). During the biotrophic stage, pathogens deploy effectors to suppress or evade host immunity and sustain the viability of host cells to increase nutrient availability for the pathogen. In the necrotrophic stage, effectors promote rapid host cell death (Koeck et al., 2011; Vleeshouwers and Oliver, 2014; Villa-Rivera et al., 2017).

Necrosis- and ethylene-inducing peptide 1-like proteins (NLPs) are a family of secreted effectors found widely in plant pathogenic oomycetes, fungi, and bacteria (Oome and Van den Ackerveken, 2014). Necrosis-and-ethylene-inducing peptide 1 (Nep1), the founding member of the family, was initially isolated from a culture filtrate of the plant pathogen *Fusarium oxysporum* (Bailey, 1995). Since the discovery of Nep1, more and more NLPs have been identified, mostly in plant-associated fungi such as *Neofusicoccum parvum* (Pour et al., 2020), *Hyaloperonospora arabidopsidis* (Lenarčič et al., 2019), *Moniliophthora perniciosa* (de Oliveira et al., 2012), *Phytophthora capsici* (Chen et al., 2018) and *Pythium oligandrum* (Yang et al., 2021). A strongly conserved heptapeptide motif (GHRHDWE) is a prominent feature of NLPs in different species (Oome and Van den Ackerveken, 2014). Based on phylogenetic analysis and the number of conserved cysteine residues, NLPs have been classified into three main types. Type 1 NLPs have two conserved cysteine residues and mainly occur in fungi, oomycetes, and a small number of bacteria. Type 2 NLPs tend to have four conserved cysteines and occur in bacteria and fungi (Oome and Van den Ackerveken, 2014; Seidl and Ackerveken, 2019). Type 3 NLPs typically contain six conserved cysteine residues, and were previously thought to occur only in Ascomycete fungi (Oome and Van den Ackerveken, 2014). However, type 3 NLPs were recently found in bacteria (Seidl and Ackerveken, 2019). Many NLPs can induce necrosis and ethylene biosynthesis in eudicots instead of monocot plants (Bailey, 1995; Lenarčič et al., 2017). However, the mechanism by which NLPs induce necrosis is unknown. Glycosylinositol phosphorylceramide (GIPC) sphingolipids located in the plasma membrane function as NLP toxin receptors in the early steps of NLP cytolysin action. Insensitivity to NLP cytolysins of monocot plants may be explained by the length of the GIPC head group and the architecture of the NLP sugar-binding site (Lenarčič et al., 2017). Research has shown that mutations in key regions cause a switch between non-toxic and toxic phenotypes within the same protein scaffold (Oome and Van den Ackerveken, 2014; Lenarčič et al., 2019).

In the genus *Colletotrichum*, six NLP identified in the *Colletotrichum higginsianum* display sequence variation in the consensus motif, and have contrasting expression profiles and necrosis-inducing activities. For example, ChNLP1 is expressed specifically at the switch to necrotrophy and is a potent cell death inducer, whereas ChNLP3 is expressed in appressoria before penetration and lacks necrosis-inducing activity (Kleemann et al., 2012). In *Colletotrichum orbiculare*, transformants constitutively expressing NLP1 failed to develop lesions in melon, but lesions were developed in *Nicotiana benthamiana* (Chen et al., 2021). Meanwhile, NLP1 lacking the signal peptide strongly induced cell death in melon but not in *N. benthamiana*. These results showed that the cell death-inducing activities and underlying mechanisms of *C. orbiculare* NLP1 differ between melon and *N. benthamiana*. For comprehensive control of anthracnose disease, it is essential to understand the mechanisms of pathogenicity. However, there have been no studies on the identification or functional characterization of NLPs from members of the *Colletotrichum acutatum* species complex.

The current study was undertaken to identify and functionally characterize NLPs in *Colletotrichum australisinense*, a member of the *C. acutatum* species complex, a hemibiotrophic pathogen of the rubber tree *Hevea brasiliensis*. Specifically, we aimed to (1) identify NLPs in *C. australisinense* and analyze their phylogenetic relationships with other known NLPs; (2) study the expression of genes encoding CaNLP in different fungal cell types *in vitro* and in the natural host (i.e., not *H. brasiliensis* infection); and (3) determine their ability to cause necrosis in host and non-host plant species.

## Materials and methods

### Identification, phylogenetic, and *in silico* analyses of necrosis- and ethylene-inducing peptide 1-like proteins

Necrosis- and ethylene-inducing peptide 1-like proteins (NLPs) and the genes encoding them were identified and categorized based on searching for the conserved motif GHRHDWE in the predicted proteins database and a TBLASTN search against the *C. australisinense* genome deposited in the National Center for Biotechnology Information (NCBI) database (accession no. JABKAN000000000.1), in which previously published NLP protein sequences are present (Kleemann et al., 2012; Oome and Van den Ackerveken, 2014). NLP sequences from the other three species belonging to *C. acutatum* species complex for which whole-genome sequencing is available, viz. *C. nymphaeae* IMI 504889 (accession no. JEMN00000000.1), *C. simmondsii* CBS 122122 (accession no. JFBX00000000.1), and *C. scovillei* TJNH1 (accession no. JAAJBS000000000.1), were retrieved using the identified NLPs of *C. australisinense* as queries against the NR database^1^ and whole-genome sequences. Signal peptides were predicted using SignalP 5 (Armenteros et al., 2019). Phylogenetic analysis was performed using MEGA 7.0 (Kumar et al., 2016). Protein sequences were aligned using MUSCLE method within MEGA 7.0 (Kumar et al., 2016) and outliers with too many gaps were removed from the analysis. A maximum likelihood tree was constructed with 1,000 bootstrap, and the final tree was visualized in FigTree v 1.3.1 (Rambaut and Drummond, 2009). Seqlogos were created with TBtools v1.082 (Chen et al., 2020). Sequence regions included the conserved cysteine residues, key residues responsible for necrosis activity, and the heptapeptide motif. Colors were set as blue for KRH, red for DE, green for STNQ, orange for FYW, pink for VLAI, purple for GP, and yellow for C.

### Heterologous expression in *Escherichia coli*

To comparatively evaluate the necrosis-inducing activity of NLPs, five CaNLPs belonging to different types were prepared as previously described (Cobos et al., 2019). CaNLP4 represents the NLP with substitution of residues known to be essential for necrosis induction activity. CaNLP5, CaNLP9, CaNLP10, and CaNLP11 contain conserved these key residues. Briefly, coding sequences (cDNAs) encoding CaNLPs proteins (without their putative signal peptides) were amplified and inserted into the *E. coli* expression vector pET SUMO to generate a His-tag fusion. Individual colonies for each construct were tested for insertions by PCR. Selected clones were verified by DNA sequencing. Expression of recombinant proteins in the *E. coli* BL21 (DE3) strain was carried out according to the manufacturer’s instructions (WeiDi, Shanghai, China). Protein expression was induced by adding 0.1 mM isopropyl-β-d-thiogalactopyranoside (IPTG) and culturing cells at 16°C for a further 8 h. Purification of His-tag fusion proteins from *E. coli* cell-free extracts was achieved by affinity purification with Ni-nitrilotriacetic acid resin (Ni-NTA; GE healthcare, Sweden) equilibrated in buffer A (50 mM NaH_2_PO_4_, 300 mM NaCl, 20 mM imidazole, pH 8). After extensive washing, bound proteins were eluted with buffer B (50 mM NaH_2_PO_4_, 300 mM NaCl, 250 mM imidazole, pH 8). Upon visual inspection following a SDS-PAGE, NLP-containing fractions were pooled and dialyzed against phosphate-buffered saline (PBS; pH 7.4) at 4°C using a Slide-A-lyzer Mini Dialysis Float System (Thermo Fisher Scientific, United States). The purity of recombinant proteins was confirmed by SDS-PAGE and quantified using Bradford method (Bradford, 1976) pre-configured in NanoDrop 2000C (Gene company, United States).

### Necrosis-inducing activity assay

Healthy and non-wounded right green leaves collected from rubber tree clones (Reyan7-33-97) were washed three times with sterilized water and dried at room temperature. *In vitro* leaves punctured by sterile syringes at the treatment site were separately dropped with 50 μL each purified NLP, or PBS buffer (pH 7.4) and proteins purified from *E coli* cells with pET SUMO empty vector (EV) as a negative control. The protein concentration was 2 μM. Each CaNLP was assayed using three replicates, and each experiment was repeated at least three times. Inoculated leaves were placed on moist tissue paper, maintained in a humidified chamber, and incubated at 25°C, with daily monitoring for lesion development. The level of leaf necrosis induced by CaNLPs was determined using ImageJ software^2^ by calculating the necrosis area at 4 dpi. Necrosis quantification was carried out by performing an electrolyte leakage assay. Five leaf discs (9 mm in diameter) were soaked in 5 ml of distilled water for 2 h at room temperature. The conductivity of the bathing solution was then measured using a TP320 conductivity meter (Timepower, Beijing, China). To analyze the specificity of different types of CaNLP, according to the method described above, we tested their necrotic-inducing activity on non-host plants including *capsicum*, the usual host of *C. scovillei*.

### Structure prediction of CaNLPs differing in cytotoxicity

Swiss-model (Biasini et al., 2014) and Phyre2 (Kelley et al., 2015) were used to predict the structures of CaNLP’s. Crystal structures of NEP-1 from *M. perniciosa* (PDB ID 3ST1) (Zaparoli et al., 2011) and a 25 kDa protein elicitor from *Pythium aphanidermatum* (PDB ID 3GNU, 3GNZ) (Ottmann et al., 2009) were used as templates to build 3D models of CaNLPs. Qmean was used for quality assessment and selection of the best model structure (Benkert et al., 2008). Structural data were visualized with Pymol version 2.5.2 (Schrodinger)^3^.

### Gene expression analysis

Different fungal growth stages were investigated. Briefly, spores from a liquid culture of *C. australisinense* were collected by filtration through a Whatman filter paper, immediately frozen in liquid nitrogen, and freeze-dried. Appressoria were collected by smearing conidia on glass paper at 24 h post-infection (hpi). Samples of infected rubber leaves were collected at 0, 24, 48, 72, and 96 h after inoculation with conidial suspension. Three biological replicates were included for each sampled fungal stage. Total RNA was extracted from two to three leaves collected from three individual plants in each experiment using an RNaprep Pure Plant Plus Kit (TIANGEN BIOTECH, Beijing, China) according to the manufacturer’s recommendations. First-strand cDNA was synthesized from 1 μg of total RNA using a FastKing RT Kit (TIANGEN BIOTECH) as the manufacturer’s protocol. To assess the expression profiles of *CaNLP* genes, RT-PCR primers (Supplementary Table 1) were designed using Primer 3.0 software (Applied Biosystems). Gene fragments ranging from 132 to 196 bp were amplified using specific primers and a T100 Thermal Cycler PCR Detection System (Bio-Rad, Singapore). Mock-inoculated *H. brasiliensis* served as a negative control for each primer pair to exclude the possibility that amplification was due to similar plant sequences. The expression changes at other different stages relative to spore were further determined by qRT-PCR (SuperReal Premix Plus; TIANGEN BIOTECH, Beijing, China) referencing Cobos’s method (2019). Briefly, qRT-PCR reactions were performed on the Quantstudio 6 Flex detection system (ABI, Singapore) under the following conditions: 95°C for 15 min; 40 cycles at 95°C for 10 s, 60°C for 30 s to calculate cycle threshold values; followed by a dissociation program of 79 cycles at 55–95°C to obtain melt curves. The threshold cycle (CT) values were determined automatically by instrument, and the fold changes of each gene were calculated by the equation 2^–ΔΔCt^. Results were obtained from three repeated trials.

## Results

### Phylogenetic analysis and classification of *Colletotrichum australisinense* necrosis- and ethylene-inducing peptide 1-like proteins

**FIGURE 1:**
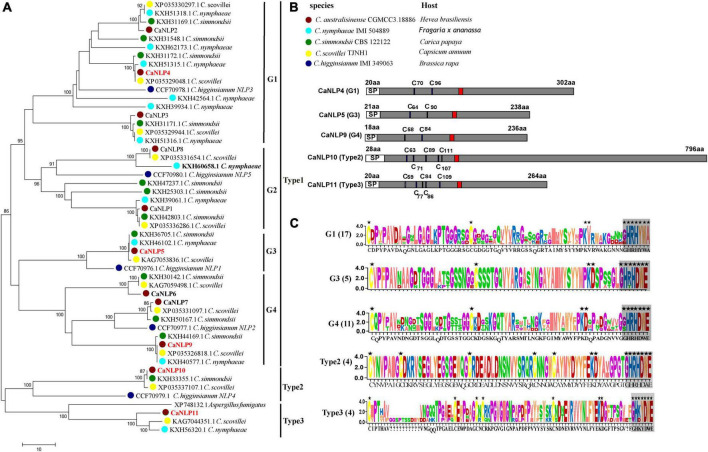
Phylogenetic relationships and sequence characteristics of all predicted Nep1-like proteins (NLPs). **(A)** Phylogenetic relationships of NLPs between *Colletotrichum australisinense* and other species in the *Colletotrichum acutatum* species complex inferred using maximum likelihood with 1,000 bootstrap replicates. Bold black NLPs lack the heptapeptide motif. Red NLPs were analyzed in necrosis-inducing activity and expression experiments. **(B)** Schematic representation of different groups and types NLP. “C” indicates the conserved cysteine residues. Red region indicates the conserved heptapeptide motif “GHRHDWE.” **(C)** Consensus sequence logos of different NLP groups and types. Black stars indicate the key residues responsible for necrosis activity, and box-shadows indicate the heptapeptide motif.

To identify NLPs in *C. australisinense*, we blasted against the whole-genome and protein dataset using the NPP1 domain as a query. We found 11 NLPs containing a NPP1 domain, hereafter referred to as CaNLP1-11, respectively. In another three species belonging to the *C. acutatum* species complex for which whole-genome sequencing data are available, viz. *C. nymphaeae* IMI 504889, *C. simmondsii* CBS 122122, and *C. scovillei* TJNH1, the number of NLPs was essentially the same as for *C. australisinense*. The amino acid sequences of 45 NLPs from the *C. acutatum* species complex were used to construct a neighbor-joining phylogenetic tree, together with five NLPs in *C. higginsianum* and a type 3 NLP in *Aspergillus fumigatus* (Oome and Van den Ackerveken, 2014). Phylogenetic analysis clearly distinguishes the diversification of *C. acutatum* species complex NLPs (Figure 1A). Based on conserved cysteine residues and the heptapeptide motif (GHRHDWE), all 51 NLPs can be divided into three types (type 1, 2, and 3). Type 1 NLPs contain two conserved cysteine residues, whereas type 2 and 3 NLPs contain five conserved cysteine residues (Figures 1B,C). We also found interspecific differences between the types of NLPs in the *C. acutatum* species complex; *C. australisinense* is the same as *C. scovillei* for all types of NLPs; however, *C. nymphaeae* lacks type 2 NLPs and *C. simmondsii* lacks type 3 NLPs. In addition, type 1 NLPs can be further divided into four groups, with high homology between members within each group (Figure 1A). Many NLPs have substitutions in the GHRHDWE heptapeptide motif; for example, group1 (G1) of type 1 NLPs have GHRHYWA at this region, whereas group2 (G2) NLPs only have two conserved residues at the second and fourth positions of the heptapeptide motif. Group3 (G3) and group4 (G4) NLPs of type 1 have the same conserved two cysteine residues and the GHRHDWE heptapeptide motif, but there are significant differences in amino acid sequences between the two groups (Figure 1C); G4 members have two additional cysteine residues in the second half of the sequence.

We further investigated type 2 NLPs of *C. australisinense*. The molecular weight of NLPs is typically < 50 kDa (Oome and Van den Ackerveken, 2014; Yang et al., 2021). However, the predicted molecular weight of CaNLP10 belonging to type 2 NLPs from *C. australisinense* was 82 kDa (Figure 1B). The BLASTp results showed that type 2 NLPs of *C. australisinense* shared 94.79% sequence identity with a putative NPP1 domain protein of *C. scovillei* (XP 035337107.1), 92.07% with a putative NPP1 domain protein from *C. simmondsii* (KXH33355.1), and 75.54% with a putative NPP1 domain type protein from *Fusarium coffeatum* (XP_031019613.1). In addition, we found that type 2 NLPs of *C. australisinense* shared homology with NPP1 family proteins of actinomycetes such as *Streptomyces* sp.

The BLASTp results also showed that type 3 NLPs of *C. australisinense* shared high sequence identity with hypothetical proteins of *C. scovillei, C. nymphaeae*, *C. fioriniae*, *C. salicis* belonging to the *C. acutatum* species complex, and *C. truncatum* belonging to the *C. truncatum* species complex. In addition, we found that it also shared high homology with NPP1 family proteins of other fungal species such as *Coniochaeta*, *Trichoderma*, *Metarhizium*, *Purpureocillium*, *Beauveria*, *Grosmannia*, *Aspergillus*, and *Fusarium*.

### Necrosis-inducing activity of CaNLPs

**FIGURE 2:**
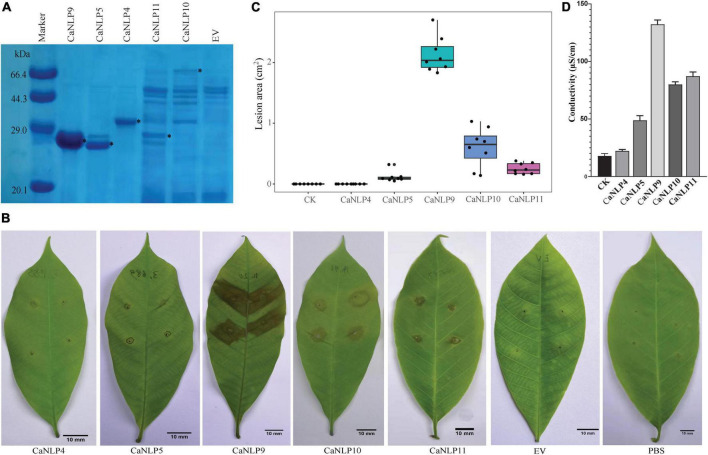
Recombinant CaNLP proteins display cytotoxic activity against *H. brasiliensis* leaves. **(A)** SDS-PAGE analysis of CaNLPs stained with Coomassie Blue. Recombinant proteins were expressed in the *E. coli* BL21 (DE3) strain using the pET SUMO vector, and partially purified using Ni-NTA resin. Black asterisks indicate target protein bands. **(B)** Representative *H. brasiliensis* leaves infiltrated with partially purified CaNLPs. Pictures were taken at 4 dpi. **(C)** Lesion areas measured by ImageJ. Data were analyzed from 10 biological repeats. **(D)** Quantification of necrotic activity induced by CaNLP proteins in plants by electrolyte leakage assay.

To assess different types of CaNLPs for their ability to induce necrosis, CaNLP4, CaNLP5, CaNLP9, CaNLP10, and CaNLP11 were selected for further studies based on NLP group/type. Genes encoding five CaNLPs without putative signal peptides were cloned into the pET SUMO expression vector and expressed in *E. coli* BL21(DE3). Expression of *CaNLPs* was verified by SDS-PAGE (Figure 2A). However, the quality of expressed *CaNLPs* differed significantly. The purified protein yield for CaNLP9 was the highest and reached 3.4 mg/ml, compared with 1.1 mg/ml for CaNLP4 and 1.0 mg/ml for CaNLP5. The purified protein yields of CaNLP10 and CaNLP11 were relatively low (0.6 and 0.8 mg/ml, respectively).

We then explored the necrosis-inducing activity of purified NLPs in host plants. For this, *H. brasiliensis* leaves punctured by sterile syringes were treated with 2 μM purified protein (or PBS as a negative control). Four out of five CaNLPs exhibited necrosis-inducing activity, and only CaNLP4 showed no necrosis-inducing activity (Figure 2B). Lesion areas and the conductivity of the necrotic tissues were measured 4 days after infiltration. The necrosis-inducing abilities of different types of CaNLPs exhibited significant differences. CaNLP9 produced the largest lesion areas on leaves at 4 dpi, while CaNLP5 caused the smallest lesions, and lesions caused by CaNLP10 and CaNLP11 were intermediate in size (Figure 2C). In addition, CaNLP10 also caused chlorotic responses in leaves. Conductivity results obtained from inoculated leaves indicated cell permeability. In subsequent electrolyte leakage assays, as expected, CaNLP9 showed the strongest cytolytic activity with the 132.2 μS/cm conductivity, CaNLP5 displayed the weakest activity with the 67.1 μS/cm conductivity, and CaNLP10 and CaNLP11 exhibited moderate cytolytic activity with the 80.0 and 87.2 μS/cm conductivity, respectively (Figure 2D).

**FIGURE 3:**
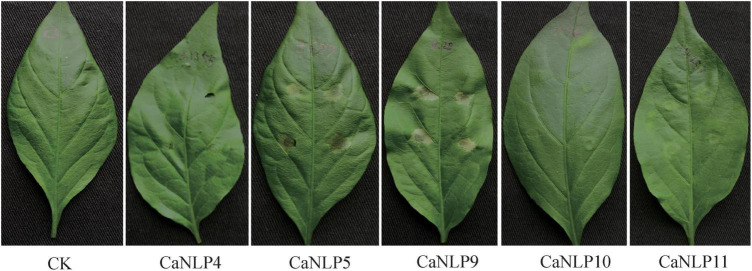
Recombinant CaNLP proteins display cytotoxic activity on *capsicum* leaves. Pictures were taken at 3 dpi.

To determine whether cytotoxic CaNLPs are specific toward *H. brasiliensis*, the usual host of *C. australisinense*, we tested the necrosis-inducing activity of purified NLPs using leaves of *capsicum*, neither of which are hosts for this pathogen. We found that CaNLP5 and CaNLP9 were capable of inducing necrosis in leaves of capsicum, while CaNLP11 only resulted in chlorotic areas, and CaNLP4 and CaNLP10 did not cause a visible reaction (Figure 3). In summary, CaNLP4 showed no cytotoxic activity against hosts or non-host plants; and CaNLP5 and CaNLP9 were capable of inducing necrosis in leaves of host and non-host plants and CaNLP10 and CaNLP11 displayed moderate toxicity against host but not non-host plants. In necrosis strength, under the same molar concentration, CaNLP9 possess the strongest necrosis-inducing activity, which caused the largest lesion areas the highest conductivity, CaNLP10 and CaNLP11 are moderate activity, and CaNLP5 is the weakest activity on treated *H. brasiliensis* leaves.

### Structural features of CaNLPs with differing toxicities

**FIGURE 4:**
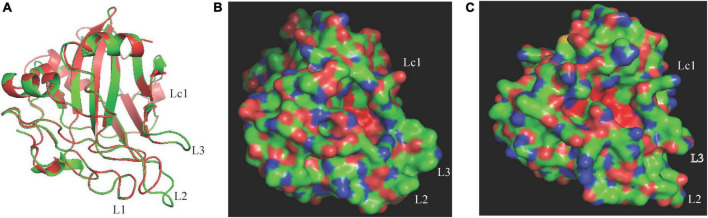
Comparison of predicted protein structure features between the weakly toxic CaNLP5 and the strongly toxic CaNLP9. **(A)** Superposition of CaNLP5 (red) and CaNLP9 (green). **(B,C)** Structures of the predicted protein surfaces of CaNLP5 and CaNLP9.

The difference in necrosis-inducing activity against *H. brasiliensis* between CaNLP5 and CaNLP9 prompted us to analyze their structural differences, hence 3D models of the two CaNLPs were predicted using crystal structures of NEP-1 from *M. perniciosa* (PDB ID 3ST1) (Zaparoli et al., 2011) and a 25 kDa protein elicitor from *P. aphanidermatum* (PDB ID 3GNU, 3GNZ) as templates. We found that the weakly toxic CaNLP5 and the strongly toxic CaNLP9 shared the same overall protein scaffold, comprising a nine-stranded β-sandwich flanked by three α-helices (Figure 4). The fold of the conserved L1–L3 loops, reported to be essential for NLP activity (Gijzen and Nurnberger, 2006; Lenarčič et al., 2019), was well-defined in both CaNLP5 and CaNLP9 models (Figure 4A). The two predicted NLP structures share a crevice located between L2 and L3, which was recently shown to be the GIPC receptor-binding motif within toxic NLPs (Lenarčič et al., 2017). One of the significant differences between CaNLP5 and CaNLP9 structures is located at the L2 loop, which is longer in CaNLP9, resulting in a larger crevice than in CaNLP5 (Figures 4B,C). Another obvious difference between the CaNLP5 and CaNLP9 structures is the conformation of the Lc1 loop (Figure 4), which affects opening of the cavity (Lenarčič et al., 2019). We also observed two small helices. Because there are no suitable protein structures as templates, 3D models of CaNLP10 and CaNLP11 were not constructed in this study.

### Expression analysis of CaNLP genes

**FIGURE 5:**
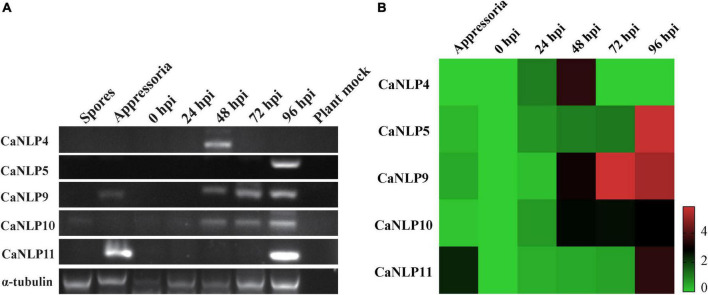
Expression analysis of CaNLP. **(A)** Detection of five *CaNLP* transcripts at spore and appressoria stages, and at 0 h, 24 h, 48, 72, and 96 hpi. **(B)** The relative expression analysis of five *CaNLPs* was determined by qRT-PCR using *tubulin* as an internal reference gene. The heatmap representing fold (log_2_) changes relative to spore expression.

To determine the transcriptional patterns of genes encoding CaNLPs in pathogens, we monitored their expression by RT-PCR in inoculated *H. brasiliensis* leaves across five time-points (0, 24, 48, 72, and 96 hpi), as well as during different developmental stages (spores and appressoria). This approach spanned the whole infection period of *H. brasiliensis* by *C. australisinense*. As shown in Figure 5A, all genes displayed elevated expression levels during the infection phases. In particular, the gene encoding the non-cytotoxic CaNLP4 was detected at the earliest infection stage (48 hpi), while the gene encoding the weakly toxic CaNLP5 was detected only at the later infection stages (96 hpi). Notably, *CaNLP11* gene transcripts were detected in appressoria and later infection stages (96 hpi), while genes encoding the strongly toxic CaNLP9 and moderately toxic CaNLP10 were induced at all infection stages (48, 72, and 96 hpi). RT-PCR analysis also confirmed that these five genes aren’t pseudo genes. The obtained qRT-PCR results showed that the gene encoding non-cytotoxic CaNLP4 was obviously upregulated expression at the 24 hpi, and reached the highest expression levels at 48 hpi, followed by a rapid decline. The expression of *CaNLP5* and *CaNLP9* was significantly upregulated expression at the 48 hpi, followed by gradually increased expression to a maximum at 96 hpi. The *CaNLP10* gene was also obviously upregulated expression at the 48 hpi, but followed by stable expression to 96 hpi. The *CaNLP11* gene was only obviously upregulated expression at the appressoria and 96 hpi (Figure 5B).

## Discussion

### Diversity and evolution of necrosis- and ethylene-inducing peptide 1-like proteins in the *Colletotrichum acutatum* species complex

Although NLPs are widely distributed in plant pathogenic oomycetes, fungi, and bacteria, there are significant differences in protein types and numbers between microbial species (Oome and Van den Ackerveken, 2014). It is estimated that only 8% of ascomycete species contain all three types of NLPs (Oome and Van den Ackerveken, 2014). In the genus *Colletotrichum*, a lineage-specific expansion of NLP families within the *C. acutatum* species complex has been reported (Baroncelli et al., 2016). In the present study, we analyzed the types and numbers of NLPs in four species belonging to the *C. acutatum* species complex. Notably, we found that *C. australisinense* and *C. scovillei* contain all three types of NLPs. This is the first report of all three types of NLPs in the same species.

It has been shown that the different types of NLPs present in microbes is associated with their lifestyle (Oome and Van den Ackerveken, 2014). Therefore, the occurrence of all three NLP types within the *C. acutatum* species complex indicates a variety of lifestyles. In the *C. acutatum* species complex, most species adopt plant-associated lifestyles (Baroncelli et al., 2017). However, *C. nymphaeae* and *C. fioriniae* are dicot plant pathogens, and are also entomopathogens infecting the insects *Praelongorthezia praelonga* and *Fiorinia externa* (Marcelino et al., 2008; Mascarin et al., 2016). We suggest that the expansion of NLPs might contribute to the broad host range of these pathogens, and horizontal gene transfer (HGT) might be the main pathway of ancient acquisition of specific types of NLPs within the *C. acutatum* species complex. This hypothesis was also proposed by Oome and Van den Ackerveken (2014), as well as Seidl and Ackerveken (2019). Conidial anastomosis tubes (CATs) have been recognized in many filamentous fungi, and were believed to be potential agents of HGT between species (Roca et al., 2005). A remarkable inter-specific and intra-specific conidia fusion through CATs was discovered in the genus *Colletotrichum* (Roca et al., 2004; Gonçalves et al., 2016; Mehta and Baghela, 2021). These results will provide important clues for understanding the evolutionary history of NLP gene family in the genus *Colletotrichum*.

### Necrosis-inducing activity of necrosis- and ethylene-inducing peptide 1-like proteins in the *Colletotrichum acutatum* species complex

The cytotoxic activity of NLPs toward plants has attracted significant attention. Several different approaches have been implemented to explore the cell death-inducing activity of purified or recombinantly produced NLPs, among which include infiltration of purified proteins into the leaf apoplast, transpiration streams upon placing leaf petioles in protein solution, spraying protein solutions as a surfactant, and Agrobacterium-mediated transient gene expression. The Agrobacterium-mediated transient gene expression method is widely used in the literature, but this method is mainly applied to model plants, such as *Nicotiana* species (Chen et al., 2021; Duhan et al., 2021). The infiltration of purified proteins method has also been used for the activity evaluation of NLPs in some forest plants (Cobos et al., 2019; Hunziker et al., 2021; Liu et al., 2021). In the present study, we employed purified recombinant protein infiltration into the leaf apoplast to analyze cytotoxic activity of NLPs, and obtained consistent results after repeated experiments. We determined that this method can also be used for activity evaluation of NLPs in *H. brasiliensis* leaves.

Cell death is a characteristic bioactivity of cytotoxic NLPs. Many microbial species also contain non-cytolytic NLPs. Several residues are known to be essential for necrosis induction activity of NLPs, including two cystine residues, the KD motif, and the heptapeptide motif GHRHDWE (Oome and Van den Ackerveken, 2014). In addition to, it has been previously confirmed that two conformations also are essential for the necrosis-inducing activity of NLPs (Lenarčič et al., 2017, 2019). In this study, we found that the non-cytolytic activity of CaNLPs resulted from substitution of the KD motif and the heptapeptide motif GHRHDWE, and changes of structure characteristics lead to significant differences in the strength of necrosis-inducing activity between CaNLPs. Similar results have been reported in the necrotrophic pathogen *Alternaria brassicae* (Duhan et al., 2021). Additionally, the responses of plants to NLPs not only depend on the type of NLP, but also the plant species in which the responses are tested. It is suggested that at least three different plant perception systems have evolved for NLPs (Seidl and Ackerveken, 2019). CaNLP10 and CaNLP11 activity displayed specificity toward *H. brasiliensis*, and NLP perception in *H. brasiliensis* differs from that in *capsicum*, suggesting that NLP perception is mediated by another receptor resulting from convergent evolution (Seidl and Ackerveken, 2019).

Expression analysis of CaNLPs revealed that genes encoding CaNLPs with different activity characteristics exhibited different expression patterns. Changes in the expression patterns of genes encoding NLPs at different stages of infection have also been reported for other *Colletotrichum* spp. (*C. higginsianum* and *C. orbiculare*) (Kleemann et al., 2012; Irieda et al., 2014). For example, the gene encoding non-cytotoxic ChNLP3 of *C. higginsianum* was expressed at 22 and 40 hpi, during preinvasive or early biotrophic pathogen growth. By contrast, genes encoding cytotoxic ChNLP1 were expressed at 60 hpi, when the pathogen switches from a biotrophic to a necrotrophic lifestyle (Kleemann et al., 2012). Cell death caused by cytotoxic NLPs could be beneficial for necrotrophic pathogens, which thrive on dead plant tissue, or for advancing to the necrotrophic stages of plant infection by hemibiotrophic pathogens. However, the exact roles of non-cytolytic NLPs in pathogen infection remain unknown, suggesting these proteins have adopted new functions as avirulence genes (Seidl and Ackerveken, 2019; Hunziker et al., 2021). In this study, all of these data indicate that these five targeted *CaNLP* genes are expressed at different levels at different times and contributed to different transcription types on the mRNA expression levels. We found that the expression of gene encoding non-cytotoxic CaNLP was negatively correlated with that of genes encoding cytotoxic CaNLP, suggesting that each targeted gene affects cells or tissues differently depending on the stage of infection (Feng et al., 2014). These results will provide a valuable reference for revealing the necrosis-inducing activity characteristics of different types NLP and understanding the pathogenic mechanism of *C. acutatum* species complex. As a next step, we will evaluate the effect of different type NLP-encoding genes knockouts to the pathogenicity of *C. australisinense*.

## Data availability statement

The datasets presented in this study can be found in online repositories. The names of the repository/repositories and accession number(s) can be found in the article/Supplementary material.

## Author contributions

XL conducted the experiments and analyzed the results. BL, JC, YY, and YF collected *Colletotrichum* isolates and performed experiments. GH revised and approved the final version of the manuscript. All authors reviewed the manuscript.

## Footnotes

1
https://www.ncbi.nlm.nih.gov/

2
https://imagej.nih.gov/ij/download.html

3
https://pymol.org/2/?#products
