# Supplementary material for: Characterization and necrosis-inducing activity of necrosis- and ethylene-inducing peptide 1-like proteins from Colletotrichum australisinense, the causative agent of rubber tree anthracnose
Source: Front Microbiol. 2022 Aug 30;13:969479. doi: 10.3389/fmicb.2022.969479 (PMC9468550; doi:10.3389/fmicb.2022.969479)
Supplement: Supplementary file 1 [file Table_1.DOCX]

**Supplementary Material**

Supplementary Table

Table S1 Oligonucleotides used in this study.

| Gene | Primer name | Primer sequence (5´–3´) | Length (bp) |
| --- | --- | --- | --- |
| CaNLP4 | CaNLP4F | GCC GTC GTT CCA TTC AC | 155 |
|  | CaNLP4R | CCA CCA CCA GTA GGC TTC A |  |
| CaNLP5 | CaNLP5F | GGC ACC GTC GGT AGC TTG T | 196 |
|  | CaNLP5R | TGG CGT AGG CTC CGT TGT A |  |
| CaNLP9F | CaNLP9F | CTC GGG AGG ATG TAA GGA C | 178 |
|  | CaNLP9R | AGC AGA ATC AAG CCA GAC G |  |
| CaNLP10F | CaNLP10F | GCGGATGACGAGTTGGAGAA | 180 |
|  | CaNLP10R | ACATTGCCAGTCGACGAGTT |  |
| CaNLP11F | CaNLP11F | AAT GAC AAC CCT CCT AAA TGA A | 132 |
|  | CaNLP11R | GGC GTT TGC TGT CCG TTG A |  |
| tubulin alpha | TubF | GCCTTCGTCCACTGGTATGT | 142 |
|  | TubR | AGTACTCGGCATCGTCCTCC |  |
